# An effective all-atom potential for proteins

**DOI:** 10.1186/1757-5036-2-2

**Published:** 2009-04-08

**Authors:** Anders Irbäck, Simon Mitternacht, Sandipan Mohanty

**Affiliations:** 1 Computational Biology & Biological Physics, Department of Theoretical Physics, Lund University, Sölvegatan 14A, SE-223 62 Lund, Sweden; 2 Jülich Supercomputing Centre, Institute for Advanced Simulation, Forschungszentrum Jülich, D-52425 Jülich, Germany

## Abstract

We describe and test an implicit solvent all-atom potential for simulations of protein folding and aggregation. The potential is developed through studies of structural and thermodynamic properties of 17 peptides with diverse secondary structure. Results obtained using the final form of the potential are presented for all these peptides. The same model, with unchanged parameters, is furthermore applied to a heterodimeric coiled-coil system, a mixed *α*/*β *protein and a three-helix-bundle protein, with very good results. The computational efficiency of the potential makes it possible to investigate the free-energy landscape of these 49–67-residue systems with high statistical accuracy, using only modest computational resources by today's standards.

**PACS Codes**: 87.14.E-, 87.15.A-, 87.15.Cc

## 1 Introduction

A molecular understanding of living systems requires modeling of the dynamics and interactions of proteins. The relevant dynamics of a protein may amount to small fluctuations about its native structure, or reorientations of its ordered parts relative to each other. In either case, a tiny fraction of the conformational space is explored. For flexible proteins, perhaps with large intrinsically disordered parts [1,2], the situation is different. When studying such proteins or conformational conversion processes like folding or amyloid aggregation, the competition between different minima on the free-energy landscape inevitably comes into focus. Studying these systems by computer simulation is a challenge, because proper sampling of all relevant free-energy minima must be ensured. This goal is very hard to achieve if explicit solvent molecules are included in the simulations. The use of coarse-grained models can alleviate this problem, but makes important geometric properties like secondary structure formation more difficult to describe.

Here we present an implicit solvent all-atom protein model especially aimed at problems requiring exploration of the global free-energy landscape. It is based on a computationally convenient effective potential, with parameters determined through full-scale thermodynamic simulations of a set of experimentally well characterized peptides. Central to the approach is the use of a single set of model parameters, independent of the protein studied. This constraint is a simple but efficient way to avoid unphysical biases, for example, toward either *α*-helical or *β*-sheet structure [3,4]. Imposing this constraint is also a way to enable systematic refinement of the potential.

**Figure 1:**
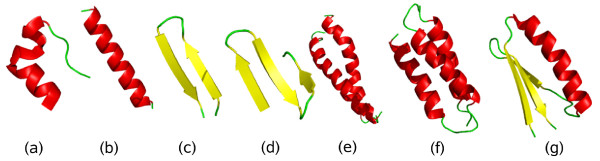
**Schematic illustration of native geometries studied**. (a) the Trp-cage, (b) an *α*-helix, (c) a *β*-hairpin, (d) a three-stranded *β*-sheet, (e) an *α*-helix dimer (1U2U), (f) a three-helix bundle (1LQ7), and (g) a mixed *α*/*β *protein (2GJH).

An earlier version [5,6] of this potential has proven useful, for example, for studies of aggregation [7-9] and mechanical unfolding [10,11]. Also, using a slightly modified form of the potential [12], the folding mechanisms of a 49-residue protein, Top7-CFr, were investigated [13,14]. Here we revise this potential, through studies of an enlarged set of 17 peptides (see Table 1 and Fig. 1). We show that the model, in its final form, folds these different sequences to structures similar to their experimental structures, using a single set of potential parameters. The description of each peptide is kept brief, to be able to discuss all systems and thereby address the issue of transferability in a direct manner. The main purpose of this study is model development rather than detailed characterization of individual systems.

**Table 1:** Amino acid sequences

System	PDB code	Sequence
Trp-cage	1L2Y	NLYIQ WLKDG GPSSG RPPPS
E6apn1	1RIJ	Ac-ALQEL LGQWL KDGGP SSGRP PPS-NH_2_
C		Ac-KETAA AKFER AHA-NH_2_
EK		Ac-YAEAA KAAEA AKAF-NH_2_
F_s_		Suc-AAAAA AAARA AAARA AAARA A-NH_2_
GCN4tp	2OVN	NYHLE NEVAR LKKLV GE
HPLC-6	1WFA	DTASD AAAAA ALTAA NAKAA AELTA ANAAA AAAAT AR-NH_2_
Chignolin	1UAO	GYDPE TGTWG
MBH12	1J4M	RGKWT YNGIT YEGR
GB1p		GEWTY DDATK TFTVT E
GB1m2		GEWTY NPATG KFTVT E
GB1m3		KKWTY NPATG KFTVQ E
trpzip1	1LE0	SWTWE GNKWT WK-NH_2_
trpzip2	1LE1	SWTWE NGKWT WK-NH_2_
betanova		RGWSV QNGKY TNNGK TTEGR
LLM		RGWSL QNGKY TLNGK TMEGR
beta3s		TWIQN GSTKW YQNGS TKIYT
AB zipper	1U2U	Ac-EVAQL EKEVA QLEAE NYQLE QEVAQ LEHEG-NH_2_
		Ac-EVQAL KKRVQ ALKAR NYALK QKVQA LRHKG-NH_2_
Top7-CFR	2GJH	ERVRI SITAR TKKEA EKFAA ILIKV FAELG YNDIN VTWDG DTVTV EGQL
GS-*α*_3 _W	1LQ7	GSRVK ALEEK VKALE EKVKA LGGGG RIEEL KKKWE ELKKK IEELG GGGEV KKVEE EVKKL EEEIK KL

Suc stands for succinylic acid.

Whether or not this potential, calibrated using data on peptides with typically ~20 residues, will be useful for larger systems is not obvious. Therefore, we also apply our potential, with unchanged parameters, to three larger systems with different geometries. These systems are the mixed *α*/*β *protein Top7-CFr, a three-helix-bundle protein with 67 residues, and a heterodimeric leucine zipper composed of two 30-residue chains.

Protein folding simulations are by necessity based on potentials whose terms are interdependent and dependent on the choice of geometric representation. Therefore, we choose to calibrate our potential directly against folding properties of whole chains. To make this feasible, we deliberately omit many details included in force fields like Amber, CHARMM and OPLS (for a review, see [15]). With this approach, we might lose details of a given free-energy minimum, but, by construction, we optimize the balance between competing minima.

Two potentials somewhat similar in form to ours are the *μ*-potential of the Shakhnovich group [16] and the PFF potential of the Wenzel group [17]. These groups also consider properties of entire chains for calibration, but use folded PDB structures or sets of decoys rather than full-scale thermodynamic simulations. Our admittedly time-consuming procedure implies that our model is trained on completely general structures, which might be an advantage when studying the dynamics of folding. Another potential with similarities to ours is that developed by the Dokholyan group for discrete molecular dynamics simulations [18].

## 2 Methods

Our model belongs to the class of implicit solvent all-atom models with torsional degrees of freedom. All geometrical parameters, like bond lengths and bond angles, are as described earlier [5].

The interaction potential is composed of four major terms:

(1)

The first term, *E*_loc_, contains local interactions between atoms separated by only a few covalent bonds. The other three terms are non-local in character: *E*_ev _represents excluded-volume effects, *E*_hb _is a hydrogen-bond potential, and *E*_sc _contains residue-specific interactions between pairs of sidechains. Next we describe the precise form of these four terms. Energy parameters are given in a unit called eu. The factor for conversion from eu to kcal/mol will be determined in the next section, by calibration against the experimental melting temperature for one of the peptides studied, the Trp-cage.

### 2.1 Local potential

The local potential  can be divided into two backbone terms,  and , and one sidechain term, . In describing the potential, the concept of a peptide unit is useful. A peptide unit consists of the backbone C*'*O group of one residue and the backbone NH group of the next residue.

• The potential  represents interactions between partial charges of neighboring peptide units along the chain. It is given by

(2)

where the outer sum runs over all pairs of nearest-neighbor peptide units and each of the two inner sums runs over atoms in one peptide unit (if the N side of the peptide unit is proline the sum runs over only C*' *and O). The partial charge *q*_*i *_is taken as ± 0.42 for C*' *and O atoms and ± 0.20 for H and N atoms. The parameter  is set to 6 eu, corresponding to a dielectric constant of *ϵ*_r _≈ 41. Two peptide units that are not nearest neighbors along the chain interact through hydrogen bonding (see below) rather than through the potential .

• The term  provides an additional OO and HH repulsion for neighboring peptide units, unless the residue flanked by the two peptide units is a glycine. This repulsion is added to make doubling of hydrogen bonds less likely. Glycine has markedly different backbone energetics compared to other residues. The lack of C_*β *_atom makes glycine more flexible. However, the observed distribution of Ramachandran *ϕ*, *ψ *angles for glycine in PDB structures [19] is not as broad as simple steric considerations would suggest.  provides an energy penalty for glycine *ψ *values around ± 120^°^, which are sterically allowed but relatively rare in PDB structures.

The full expression for  is

(3)

where  = 1.2 eu,  = -0.15 eu, *I *is a residue index, and

(4)

(5)

(6)

The function *f*(*u*_*I*_) is positive if the H_*I *_H_*I*+1 _distance, *d*(H_*I*_, H_*I*+1_), is smaller than both of the H_*I *_N_*I*+1 _and N_*I *_H_*I*+1 _distances, and zero otherwise. This term thus provides an energy penalty when H_*I *_and H_*I*+1 _are exposed to each other (it is omitted if residue *I *or *I *+ 1 is a proline). Similarly, *f*(*v*_*I*_) is positive when O_*I *_and O_*I*+1 _are exposed to each other.

•  is an explicit torsion angle potential for sidechain angles, *χ*_*i*_. Many sidechain angles display distributions resembling what one would expect based on simple steric considerations. The use of the torsion potential is particularly relevant for *χ*_2 _in asparagine and aspartic acid and *χ*_3 _in glutamine and glutamic acid. The torsion potential is defined as

(7)

where  and *n*_*i *_are constants. Each sidechain angle *χ*_*i *_belongs to one of four classes associated with different values of  and *n*_*i *_(see Table 2).

**Table 2:** Classification of sidechain angles, *χ*_*i*_

Residue	*χ*_1_	*χ*_2_	*χ*_3_	*χ*_4_
Ser, Cys, Thr, Val	I			
Ile, Leu	I	I		
Asp, Asn	I	IV		
His, Phe, Tyr, Trp	I	III		
Met	I	I	II	
Glu, Gln	I	I	IV	
Lys	I	I	I	I
Arg	I	I	I	III

The parameters of the torsion angle potential  are (, *n*_*i*_) = (0.6 eu, 3) for class I, (, *n*_*i*_) = (0.3 eu, 3) for class II, (, *n*_*i*_) = (0.4 eu, 2) for class III, and (, *n*_*i*_) = (-0.4 eu, 2) for class IV.

### 2.2 Excluded volume

Excluded-volume effects are modeled using the potential

(8)

where the summation is over all pairs of atoms with a non-constant separation, *κ*_ev _= 0.10 eu, and *σ*_*i *_= 1.77, 1.75, 1.53, 1.42 and 1.00 Å for S, C, N, O and H atoms, respectively. The parameter *λ*_*ij *_is unity for pairs connected by three covalent bonds and *λ*_*ij *_= 0.75 for all other pairs. To speed up the calculations, *E*_ev _is evaluated using a cutoff of 4.3 *λ*_*ij *_Å.

### 2.3 Hydrogen bonding

Our potential contains an explicit hydrogen-bond term, *E*_hb_. All hydrogen bonds in the model are between NH and CO groups. They connect either two backbone groups or a charged sidechain (aspartic acid, glutamic acid, lysine, arginine) with a backbone group. Two neighboring peptide units, which interact through the local potential (see above), are not allowed to hydrogen bond with each other.

The form of the hydrogen-bond potential is

(9)

where  = 3.0 eu and  = 2.3 eu set the strengths of backbone-backbone and sidechain-backbone bonds, respectively, *r*_*ij *_is the HO distance, *α*_*ij *_is the NHO angle, and *β*_*ij *_is the HOC angle. The functions *u*(*r*) and *v*(*α*, *β*) are given by

(10)

(11)

where *σ*_hb _= 2.0 Å. A 4.5 Å cutoff is used for *u*(*r*).

### 2.4 Sidechain potential

Our sidechain potential is composed of two terms, *E*_sc _= *E*_hp _+ *E*_ch_. The *E*_ch _term represents interactions among sidechain charges. The first and more important term, *E*_hp_, is meant to capture the effects of all other relevant interactions, especially effective hydrophobic attraction. For convenience, *E*_hp _and *E*_ch _have a similar form,

(12)

Here the sums run over residue pairs *IJ*,  and  are contact measures that take values between 0 and 1, and  and  are energy parameters.

It is assumed that ten of the twenty natural amino acids contribute to *E*_hp_, see Table 3. Included among these ten are lysine and arginine, which are charged but have large hydrophobic parts. To reduce the number of parameters, the hydrophobic contact energies are taken to be additive,  = *m*_*I *_+ *m*_*J *_. It is known that the statistically derived Miyazawa-Jernigan contact matrix [20] can be approximately decomposed this way [21]. The *m*_*I *_parameters can be found in Table 3.  is set to 0 if residues *I *and *J *are nearest neighbors along the chain, and is reduced by a factor 2 for next-nearest neighbors.

**Table 3:** The parameter *m*_*I *_of the hydrophobicity potential *E*_hp_

Residue	*m*_*I *_(eu)
Arg	0.3
Met, Lys	0.4
Val	0.6
Ile, Leu, Pro	0.8
Tyr	1.1
Phe, Trp	1.6

The residues taken as charged are aspartic acid, glutamic acid, lysine and arginine. The charge-charge contact energy is –  = 1.5*s*_*I *_*s*_*J *_eu, where *s*_*I *_and *s*_*J *_are the signs of the charges (± 1).

The contact measure  is calculated using a predetermined set of atoms for each amino acid, denoted by  (see Table 4). Let *n*_*I *_be the number of atoms in  and let

**Table 4:** Atoms used in the calculation of the contact measure

Residue	Set of atoms (*A*_*I*_)
Pro	C_*β*_, C_*γ*_, C_*δ*_
Tyr	C_*γ*_, C_*δ*1_, C_*δ*2_, C_*ϵ*1_, C_*ϵ*2_, C_*ζ*_
Val	C_*β*_, C_*γ*1_, C_*γ*2_
Ile	C_*β*_, C_*γ*1_, C_*γ*2_, C_*δ*_
Leu	C_*β*_, C_*γ*_, C_*δ*1_, C_*δ*2_
Met	C_*β*_, C_*γ*_, S_*δ*_, C_*ϵ*_
Phe	C_*γ*_, C_*δ*1_, C_*δ*2_, C_*ϵ*1_, C_*ϵ*2_, C_*ζ*_
Trp	C_*γ*_, C_*δ*1_, C_*δ*2_, C_*ϵ*3_, C_*ζ*3_, C_*η*2_
Arg	C_*β*_, C_*γ*_
Lys	C_*β*_, C_*γ*_, C_*δ*_

(13)

where *g*(*x*) is unity for *x *< (3.7 Å)^2^, vanishes for *x *> (4.5 Å)^2^, and varies linearly for intermediate *x*. The contact measure can then be written as

(14)

where *γ*_*IJ *_is either 1 or 0.75. For *γ*_*IJ *_= 1,  is, roughly speaking, the fraction of atoms in  and  that are in contact with some atom from the other of the two sets. A reduction to *γ*_*IJ *_= 0.75 makes it easier to achieve a full contact ( = 1). The value *γ*_*IJ *_= 0.75 is used for interactions within the group proline, phenylalanine, tyrosine and tryptophan, to make face-to-face stacking of these sidechains less likely. It is also used within the group isoleucine, leucine and valine, because a full contact is otherwise hard to achieve for these pairs. In all other cases, *γ*_*IJ *_is unity.

The definition of  is similar. The *γ*_*IJ *_parameter is unity for charge-charge interactions, and the sets of atoms used, , can be found in Table 5.

**Table 5:** Atoms used in the calculation of the contact measure

Residue	Set of atoms (*A*_*I*_)
Arg	N_*ϵ*_, C_*ζ*_, N_*η*1_, N_*η*2_
Lys	^1^H_*ζ*_, ^2^H_*ζ*_, ^3^H_*ζ*_
Asp	O_*δ*1_, O_*δ*2_
Glu	O_*ϵ*1_, O_*ϵ*2_

### 2.5 Chain ends

Some of the sequences we study have extra groups attached at one or both ends of the chain. The groups occurring are N-terminal acetyl and succinylic acid, and C-terminal NH_2_. When such a unit is present, the model assumes polar NH and CO groups beyond the last C_*α *_atom to hydrogen bond like backbone NH/CO groups but with the strength reduced by a factor 2 (multiplicatively). The charged group of succinylic acid interacts like a charged sidechain.

In the absence of end groups, the model assumes the N and C termini to be positively and negatively charged, respectively, and to interact like charged sidechains.

### 2.6 Monte Carlo details

We investigate the folding thermodynamics of this model by Monte Carlo (MC) methods. The simulations are done using either simulated tempering (ST) [22,23] or parallel tempering/replica exchange (PT) [24,25], both with temperature as a dynamical variable. For small systems we use ST, with seven geometrically distributed temperatures in the range 279 K–367 K. For each system, ten independent ST runs are performed. For our largest systems we use PT with a set of sixteen temperatures, spanning the same interval. Using fourfold multiplexing [26], one run comprising 64 parallel trajectories is performed for each system. The PT temperature distribution is determined by an optimization procedure [26]. The length of our different simulations can be found in Table 6.

**Table 6:** Algorithm used and total number of elementary MC steps for all systems studied

System	Method	MC steps
Trp-cage, E6apn1	ST	10 × 1.0 × 10^9^
C, EK, F_s_, GCN4tp	ST	10 × 1.0 × 10^9^
HPLC-6	ST	10 × 3.0 × 10^9^
Chignolin	ST	10 × 0.5 × 10^9^
MBH12	ST	10 × 1.0 × 10^9^
GB1p	ST	10 × 2.0 × 10^9^
GB1m2, GB1m3	ST	10 × 1.0 × 10^9^
Trpzip1, trpzip2	ST	10 × 1.0 × 10^9^
betanova, LLM	ST	10 × 1.0 × 10^9^
beta3s	ST	10 × 2.0 × 10^9^
AB zipper	PT	64 × 3.0 × 10^9^
Top7-CFR	PT	64 × 2.4 × 10^9^
GS-*α*_3 _W	PT	64 × 3.5 × 10^9^

Three different conformational updates are used in the simulations: single variable updates of sidechain and backbone angles, respectively, and Biased Gaussian Steps (BGS) [27]. The BGS move is semi-local and updates up to eight consecutive backbone degrees of freedom in a manner that keeps the ends of the segment approximately fixed. The ratio of sidechain to backbone updates is the same at all temperatures, whereas the relative frequency of the two backbone updates depends on the temperature. At high temperatures the single variable update is the only backbone update used, and at low temperatures only BGS is used. At intermediate temperatures both updates are used.

The AB zipper, a two-chain system, is studied using a periodic box of size (158 Å)^3^. In addition to the conformational updates described above, the simulations of this system used rigid body translations and rotations of individual chains.

Our simulations are performed using the open source C++-package PROFASI [28]. Future public releases of PROFASI will include an implementation of the force field described here. While this force field has been implemented in PROFASI in an optimized manner, this optimization does not involve a parallel evaluation of the potential on many processors. Therefore, in our simulations the number of processors used is the same as the number of MC trajectories generated. For a typical small peptide, a trajectory of the length as given in Table 6 takes ~18 hours to generate on an AMD Opteron processor with ~2.0 GHz clock rate. For the largest system studied, GS-*α*_3 _W, the simulations, with a proportionately larger number of MC updates, take ~10 days to complete.

### 2.7 Analysis

In our simulations, we monitor a variety of different properties. Three important observables are as follows.

1. *α*-helix content, *h*. A residue is defined as helical if its Ramachandran angle pair is in the region -90° <*ϕ *< -30°, -77° <*ψ *< -17°. Following [29], a stretch of *n *> 2 helical residues is said to form a helical segment of length *n *- 2. For an end residue that is not followed by an extra end group, the (*ϕ*, *ψ*) pair is poorly defined. Thus, for a chain with *N *residues, the maximum length of a helical segment is *N *- 4, *N *- 3 or *N *- 2, depending on whether there are zero, one or two end groups. The *α*-helix content *h *is defined as the total length of all helical segments divided by this maximum length.

2. Root-mean-square deviation from a folded reference structure, bRMSD/RMSD/pRMSD. bRMSD is calculated over backbone atoms, whereas RMSD is calculated over all heavy atoms. All residues except the two end residues are included in the calculation, unless otherwise stated. For the case of the dimeric AB zipper, the periodic box used for the simulations has to be taken into account. The two chains in the simulation might superficially appear to be far away when they are in fact close, because of periodicity. For this case we evaluate backbone RMSD over atoms taken from both chains in the dimer, and minimize this value with respect to periodic translations. We denote this as pRMSD.

3. Nativeness measure based on hydrogen bonds, *q*_hb_. This observable has the value 1 if at most two native backbone-backbone hydrogen bonds are missing, and is 0 otherwise. A hydrogen bond is considered formed if its energy is less than -1.03 eu.

In many cases, it turns out that the temperature dependence of our results can be approximately described in terms of the simple two-state model

(15)

where *X*(*T*) is the quantity studied, *X*_1 _and *X*_2 _are the values of *X *in the two states, and *K*(*T*) is the effective equilibrium constant (*R *is the gas constant). In this first-order form, *K*(*T*) contains two parameters: the melting temperature *T*_m _and the energy difference Δ*E*. The parameters *T*_m_, Δ*E*, *X*_1 _and *X*_2 _are determined by fitting to data.

Thermal averages and their statistical errors are calculated by using the jackknife method [30], after discarding the first 20% of each MC trajectory for thermalization.

Figures of 3D structures were prepared using PyMOL [31].

## 3 Results

We study a total of 20 peptide/protein systems, listed in Table 1 (amino acid sequences can be found in this table). Among these, there are 17 smaller systems with 10–37 residues and 3 larger ones with ≥ 49 residues. Many of the smaller systems have been simulated by other groups, in some cases with explicit water (for a review, see [32]). Two of the three larger systems, as far as we know, have not been studied using other force fields. A study of the 67-residue three-helix-bundle protein GS-*α*_3 _W using the ECEPP/3 force field was recently reported [33]. The simulations presented here use the same geometric representation and find about a hundred times the number of independent folding events, while consuming much smaller computing resources.

### 3.1 Trp-cage and E6apn1

The Trp-cage is a designed 20-residue miniprotein with a compact helical structure [34]. Its NMR-derived native structure (see Fig. 1) contains an *α*-helix and a single turn of 3_10_-helix [34]. The E6apn1 peptide was designed using the Trp-cage motif as a scaffold, to inhibit the E6 protein of papillomavirus [35]. E6apn1 is three residues larger than the Trp-cage but has a similar structure, except that the *α*-helix is slightly longer [35].

As indicated earlier, we use melting data for the Trp-cage to set the energy scale of the model. For this peptide, several experiments found a similar melting temperature, *T*_m _~315 K [34,36,37]. In our model, the heat capacity of the Trp-cage displays a maximum at *RT *= 0.4722 ± 0.0008 eu. Our energy unit eu is converted to kcal/mol by setting this temperature equal to the experimental melting temperature (315 K). Having done that, there is no free parameter left in the model. Other systems are thus studied without tuning any model parameter. For E6apn1, the experimental melting temperature is *T*_m _~305 K [35].

**Figure 2:**
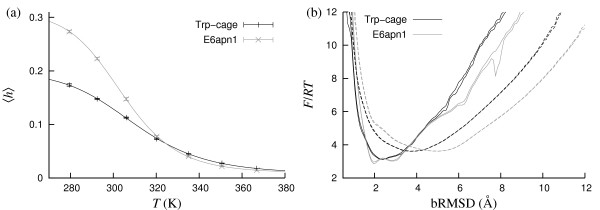
**The Trp-cage and E6apn1**. (a) Helix content *h *against temperature. The lines are two-state fits (*T*_m _= 309.6 ± 0.7 K and Δ*E *= 11.3 ± 0.3 kcal/mol for the Trp-cage; *T*_m _= 304.0 ± 0.5 K and Δ*E *= 14.2 ± 0.3 kcal/mol for E6apn1). (b) Free energy *F *calculated as a function of bRMSD at two different temperatures, 279 K (solid lines) and 306 K (dashed lines). The double lines indicate the statistical errors.

Fig. 2a shows the helix content *h *against temperature for the Trp-cage and E6apn1, as obtained from our simulations. In both cases, the *T *dependence is well described by the simple two-state model of Eq. 15. The fitted melting temperatures are *T*_m _= 309.6 ± 0.7 K and *T*_m _= 304.0 ± 0.5 K for the Trp-cage and E6apn1, respectively. This *T*_m _value for the Trp-cage is slightly lower than that we obtain from heat capacity data, 315 K. A fit to our data for the hydrophobicity energy *E*_hp _(not shown) gives instead a slightly larger *T*_m_, 321.1 ± 0.8 K. This probe dependence of *T*_m _implies an uncertainty in the determination of the energy scale. By using the Trp-cage, this uncertainty is kept small (~2%). For many other peptides, the spread in *T*_m _is much larger (see below).

Fig. 2b shows the free energy calculated as a function of bRMSD for the Trp-cage and E6apn1 at two different temperatures. The first temperature, 279 K, is well below *T*_m_. Here native-like conformations dominate and the global free-energy minima are at 2.4 Å and 2.0 Å for the Trp-cage and E6apn1, respectively. At the second temperature, 306 K, the minima are shifted to higher bRMSD. Note that these free-energy profiles, taken near *T*_m_, show no sign of a double-well structure. Hence, these peptides do not show a genuine two-state behavior in our simulations, even though the melting curves (Fig. 2a) are well described by a two-state model, as are many experimentally observed melting curves.

### 3.2 The *α*-helices C, EK, F_s_, GCN4tp and HPLC-6

Our next five sequences form *α*-helices. Among these, there are large differences in helix stability, according to CD studies. The least stable are the C [38] and EK [39] peptides, which are only partially stable at *T *~273 K. The original C peptide is a 13-residue fragment of ribonuclease A, but the C peptide here is an analogue with two alanine substitutions and a slightly increased helix stability [40]. The EK peptide is a designed alanine-based peptide with 14 residues.

Our third *α*-helix peptide is the 21-residue F_s _[41], which is also alanine-based. F_s _is more stable than C and EK [41,42], with estimated *T*_m _values of 308 K [42] and 303 K [43] from CD studies and 334 K from an IR study [44]. Even more stable is HPLC-6, a winter flounder antifreeze peptide with 37 residues. CD data suggest that the helix content of HPLC-6 remains non-negligible, ~0.10, at temperatures as high as ~343 K [45]. Our fifth helix-forming sequence, which we call GCN4tp, has 17 residues and is taken from a study of GCN4 coiled-coil formation [46]. Its melting behavior has not been studied, as far as we know, but its structure was characterized by NMR [46].

**Figure 3:**
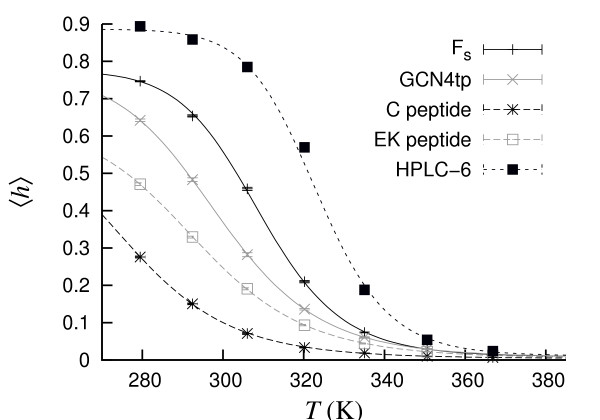
**The C, EK, F_s_, GCN4tp and HPLC-6 peptides**. Helix content *h *against temperature. The lines are two-state fits (*T*_m _= 276.3 ± 2.4 K and Δ*E *= 11.7 ± 0.4 kcal/mol for C; *T*_m _= 293.9 ± 0.4 K and Δ*E *= 12.6 ± 0.2 kcal/mol for EK; *T*_m _= 309.2 ± 0.3 K and Δ*E *= 18.7 ± 0.4 kcal/mol for F_s_; *T*_m _= 298.9 ± 0.1 K and Δ*E *= 14.1 ± 0.1 kcal/mol for GCN4tp; *T*_m _= 323.3 ± 1.2 K and Δ*E *= 23.6 ± 2.2 kcal/mol for HPLC-6).

These five peptides are indeed *α*-helical in our model. At 279 K, the calculated helix content *h *is 0.28 for the C peptide, 0.47 for the EK peptide, and > 0.60 for the other three peptides. Fig. 3 shows the temperature dependence of *h*. By fitting Eq. 15 to the data for the three stable sequences, we find melting temperatures of 298.9 ± 0.1 K, 309.2 ± 0.3 K and 323.3 ± 1.2 K for GNC4tp, F_s _and HPLC-6, respectively.

For the four peptides whose melting behavior has been studied experimentally, these results are in good agreement with experimental data. In particular, we find that HPLC-6 indeed is more stable than F_s _in the model, which in turn is more stable than both C and EK. The model thus captures the stability order among these peptides.

### 3.3 The *β*-hairpins chignolin and MBH12

We now turn to *β*-sheet peptides and begin with the *β*-hairpins chignolin [47] and MBH12 [48] with 10 and 14 residues, respectively. Both are designed and have been characterized by NMR. For chignolin, *T*_m _values in the range 311–315 K were reported [47], based on CD and NMR. We are not aware of any melting data for MBH12.

**Figure 4:**
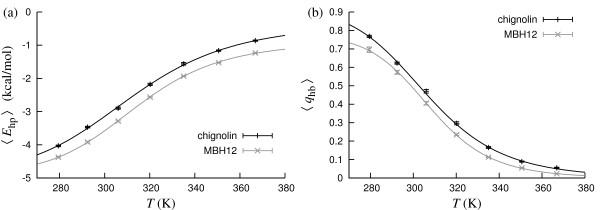
**Chignolin and MBH12**. (a) Hydrophobicity energy *E*_hp _against temperature. The lines are two-state fits (*T*_m _= 311.0 ± 0.5 K and Δ*E *= 9.6 ± 0.2 kcal/mol for chignolin; *T*_m _= 315.4 ± 1.3 K and Δ*E *= 9.9 ± 0.9 kcal/mol for MBH12). (b) Nativeness *q*_hb _against temperature. The lines are two-state fits (*T*_m _= 305.4 ± 0.5 K and Δ*E *= 10.4 ± 0.1 kcal/mol for chignolin; *T*_m _= 309.2 ± 0.7 K and Δ*E *= 13.5 ± 0.2 kcal/mol for MBH12).

Fig. 4 shows the temperature dependence of the hydrophobicity energy *E*_hp _and the nativeness parameter *q*_hb _for these peptides. By fitting to *E*_hp _data, we obtain *T*_m _= 311.0 ± 0.5 K and *T*_m _= 315.4 ± 1.3 K for chignolin and MBH12, respectively. Using *q*_hb _data instead, we find *T*_m _= 305.4 ± 0.5 K for chignolin and *T*_m _= 309.2 ± 0.7 K for MBH12. These *T*_m _values show a significant but relatively weak probe dependence. The values for chignolin can be compared with experimental data, and the agreement is good.

Because these peptides have only four native hydrogen bonds each, one may question our definition of *q*_hb _(see Methods), which takes a conformation as native-like (*q*_hb _= 1) even if two hydrogen bonds are missing. Therefore, we repeated the analysis using the stricter criterion that native-like conformations (*q*_hb _= 1) may lack at most one hydrogen bond. The resulting decrease in native population, as measured by the average *q*_hb_, was ~0.1 or smaller at all temperatures. Even with this stricter definition, we find native populations well above 0.5 at low temperatures for both peptides.

### 3.4 The *β*-hairpins GB1p, GB1m2 and GB1m3

GB1p is the second *β*-hairpin of the B1 domain of protein G (residues 41–56). Its folded population has been estimated by CD/NMR to be 0.42 at 278 K [49] and ~0.30 at 298 K [50], whereas a Trp fluorescence study found a *T*_m _of 297 K [51], corresponding to a somewhat higher folded population. GB1m2 and GB1m3 are two mutants of GB1p with significantly enhanced stability [50]. At 298 K, the folded population was found to be 0.74 ± 0.05 for GB1m2 and 0.86 ± 0.03 for GB1m3, based on CD and NMR measurements [50]. It was further estimated that *T*_m _= 320 ± 2 K for GB1m2 and *T*_m _= 333 ± 2 K for GB1m3 [50].

All these three peptides are believed to adopt a structure similar to that GB1p has as part of the protein G B1 domain (PDB code 1GB1). This part of the full protein contains seven backbone-backbone hydrogen bonds. These hydrogen bonds are the ones we consider when evaluating *q*_hb _for these peptides.

**Figure 5:**
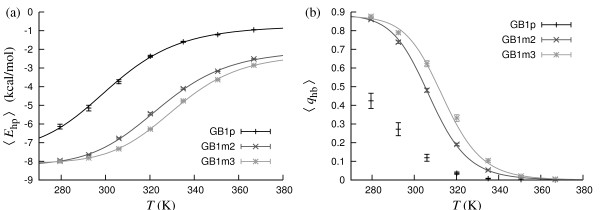
**GB1p, GB1m2 and GB1m3**. (a) Hydrophobicity energy *E*_hp _against temperature. The lines are two-state fits (*T*_m _= 301.7 ± 3.3 K and Δ*E *= 11.3 ± 1.1 kcal/mol for GB1p; *T*_m _= 324.4 ± 1.4 K and Δ*E *= 13.2 ± 1.0 kcal/mol for GB1m2; *T*_m _= 331.4 ± 0.7 K and Δ*E *= 14.8 ± 0.5 kcal/mol for GB1m3). (b) Nativeness *q*_hb _against temperature. The lines are two-state fits (*T*_m _= 307.5 ± 0.5 K and Δ*E *= 20.7 ± 0.5 kcal/mol for GB1m2; *T*_m _= 313.9 ± 1.4 K and Δ*E *= 21.4 ± 1.1 kcal/mol for GB1m3).

Fig. 5 shows the observables *E*_hp _and *q*_hb _against temperature for these peptides. Fits to the data give *E*_hp_-based *T*_m _values of 301.7 ± 3.3 K, 324.4 ± 1.1 K and 331.4 ± 0.7 K for GB1p, GB1m2 and GB1m3, respectively, and *q*_hb_-based *T*_m _values of 307.5 ± 0.5 K and 313.9 ± 1.4 K for GB1m2 and GB1m3, respectively. The *q*_hb _data do not permit a reliable fit for the less stable GB1p. At 298 K, we find *q*_hb_-based folded populations of 0.20, 0.64 and 0.74 for GB1p, GB1m2 and GB1m3, respectively, which can be compared with the above-mentioned experimental results (0.30, 0.74 and 0.86).

These results show that, in the model, the apparent folded populations of these peptides depend quite strongly on the observable studied. Our *E*_hp_-based results agree quite well with experimental data, especially for GB1m2 and GB1m3, whereas our *q*_hb _results consistently give lower folded populations for all peptides. The stability order is the same independent of which of the two observables we study, namely GB1p < GB1m2 < GB1m3, which is the experimentally observed order.

The stability difference between GB1m2 and GB1m3 is mainly due to charge-charge interactions. In our previous model [6], these interactions were ignored, and both peptides had similar stabilities. The present model splits this degeneracy. Moreover, the magnitude of the splitting, which sensitively depends on the strength of the charge-charge interactions, is consistent with experimental data.

### 3.5 The *β*-hairpins trpzip1 and trpzip2

The 12-residue trpzip1 and trpzip2 are designed *β*-hairpins, each containing two tryptophans per *β*-strand [52]. The only difference between the two sequences is a transposition of an aspargine and a glycine in the hairpin turn. CD measurements suggest that trpzip1 and trpzip2 are remarkably stable for their size, with *T*_m _values of 323 K and 345 K, respectively [52]. A complementary trpzip2 study, using both experimental and computational methods, found *T*_m _values to be strongly probe-dependent [53].

**Figure 6:**
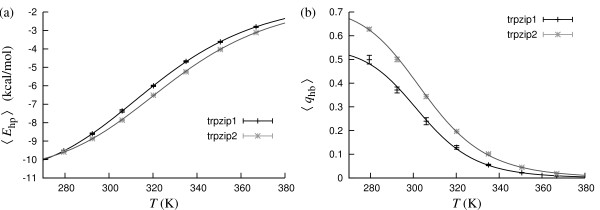
**Trpzip1 and trpzip2**. (a) Hydrophobicity energy *E*_hp _against temperature. The lines are two-state fits (*T*_m _= 319.7 ± 0.2 K and Δ*E *= 7.9 ± 0.1 kcal/mol for trpzip1; *T*_m _= 327.1 ± 0.8 K and Δ*E *= 8.3 ± 0.4 kcal/mol for trpzip2). (b) Nativeness *q*_hb _against temperature. The lines are two-state fits (*T*_m _= 303.2 ± 1.8 K and Δ*E *= 14.1 ± 0.5 kcal/mol for trpzip1; *T*_m _= 305.0 ± 1.1 K and Δ*E *= 12.6 ± 0.3 kcal/mol for trpzip2).

Fig. 6 shows our melting curves for these peptides, based on the observables *E*_hp _and *q*_hb_. The *E*_hp_-based *T*_m _values are 319.7 ± 0.2 K and 327.1 ± 0.8 K for trpzip1 and trpzip2, respectively. Using *q*_hb _data instead, we find *T*_m _= 303.2 ± 1.1 K for trpzip1 and *T*_m _= 305.0 ± 1.1 K for trpzip2.

Like for the other *β*-hairpins discussed earlier, our *q*_hb_-based folded populations are low compared to estimates based on CD data, whereas those based on *E*_hp _are much closer to experimental data. For trpzip2, the agreement is not perfect but acceptable, given that *T*_m _has been found to be strongly probe-dependent for this peptide [53].

### 3.6 Three-stranded *β*-sheets: betanova, LLM and beta3s

Betanova [54], the betanova triple mutant LLM [55] and beta3s [56] are designed 20-residue peptides forming three-stranded *β*-sheets. All the three peptides are marginally stable. NMR studies suggest that the folded population at 283 K is 0.09 for betanova [55], 0.36 for LLM [55], and 0.13–0.31 for beta3s [56].

**Figure 7:**
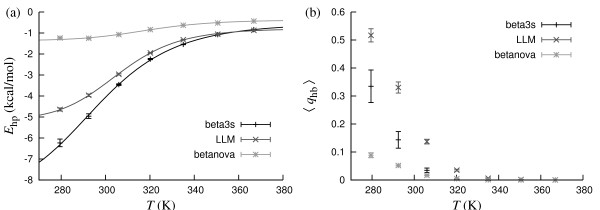
**Betanova, LLM and beta3s**. (a) Hydrophobicity energy *E*_hp _against temperature. The lines are two-state fits (*T*_m _= 318.8 ± 2.5 K and Δ*E *= 13.3 ± 2.1 kcal/mol for betanova; *T*_m _= 305.6 ± 1.7 K and Δ*E *= 13.4 ± 1.0 kcal/mol for LLM; *T*_m _= 295.7 ± 3.1 K and Δ*E *= 9.7 ± 0.5 kcal/mol for beta3s). (b) Nativeness *q*_hb _against temperature. Two-state fits were not possible.

Fig. 7 shows our *E*_hp _and *q*_hb _data for these peptides. From the *q*_hb _data, *T*_m _values cannot be extracted, because the stability of the peptides is too low. At 283 K, the *q*_hb_-based folded populations are 0.08, 0.47, 0.28 for betanova, LLM and beta3s, respectively, in good agreement with the experimental results. Fits to *E*_hp _data can be performed. The obtained *T*_m _values are 318.8 ± 2.5 K, 305.6 ± 1.7 K and 295.7 ± 3.1 K for betanova, LLM and beta3s, respectively.

These *E*_hp_-based *T*_m _values are high compared to the experimentally determined folded populations, especially for betanova. Note that betanova has a very low hydrophobicity. The correlation between *E*_hp _and folding status is therefore likely to be weak for this peptide.

In contrast to the *E*_hp_-based folded populations, those based on *q*_hb _agree quite well with experimental data. In this respect, the situation is the opposite to what we found for the *β*-hairpins studied above. A possible reason for this difference is discussed below.

### 3.7 AB zipper

The AB zipper is a designed heterodimeric leucine zipper, composed of an acidic A chain and a basic B chain, each with 30 residues [57]. The dimer structure has been characterized by NMR, and a melting temperature of ~340 K was estimated by CD measurements (at neutral pH) [57].

**Figure 8:**
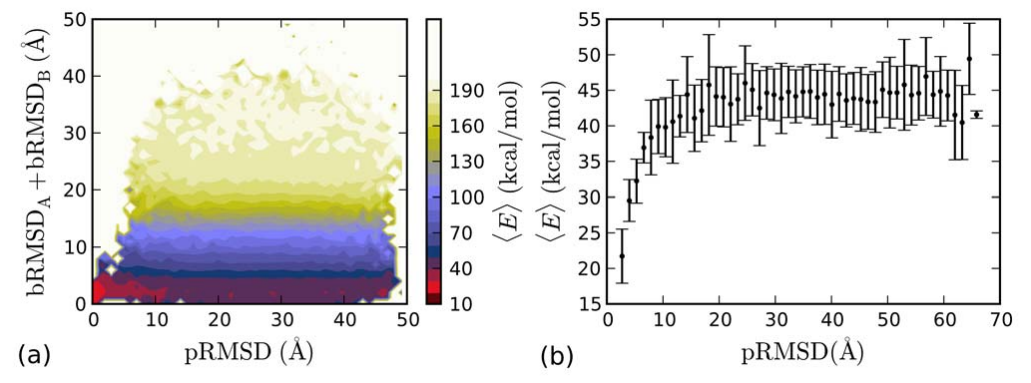
**The heterodimeric AB zipper**. (a) Mean energy as a function of pRMSD over both chains and the sum of individual bRMSDs. The direction of the energy gradients implies that a system with two folded monomers is energetically favorable compared to unfolded monomers. The proper dimeric form is the area closest to the origin, and has a lower energy. (b) Mean energy of all states in which both chains have bRMSD < 5 Å, shown as a function of the dimer RMSD measure pRMSD.

The lowest energy state seen in our simulations is a conformation in which pRMSD calculated over backbone atoms of all residues in both chains is ~2.7 Å. In this structure, the bRMSD (all residues) of the individual chains A and B to their counterparts in the PDB structure are ~2.5 Å and ~2.4 Å, respectively. Unlike for the other systems described in this article, the boundary conditions have a non-trivial role for this dimeric system. A proper discussion of periodicity, concentration and temperature dependence of this system is beyond the scope of this article. In Fig. 8a, we show the energy landscape, i.e., the mean energy as a function of two order parameters for this system. The X-axis shows the measure pRMSD described earlier. The Y-axis represents the sum of the backbone RMSD of the individual chains. pRMSD can be very large even if the sum of bRMSDs is small: the two chains can be folded without making the proper interchain contacts. Indeed, the figure shows that the major energy gradients are along the Y-axis, showing that it is energetically favorable for both chains to fold to their respective helical states. The correct dimeric native state is energetically more favorable by ~20 kcal/mol compared to two folded helices without proper interchain contacts. This is seen more clearly in Fig. 8b, where we plot the average energy as a function of pRMSD for states with two folded chains. We also simulated the two chains A and B of the dimer in isolation. Both chains folded to their native helical conformations. The melting temperatures estimated based on helix content for chains A and B are 314 K and 313 K, respectively. As indicated above, for the dimer, thermodynamic parameters like *T*_m _cannot be directly estimated from the present simulations.

### 3.8 Top7-CFr

Top7-CFr, the C-terminal fragment of the designed 93-residue *α*/*β*-protein Top7 [58], is the most complex of all molecules studied here. It has both *α*-helix and *β*-strand secondary structure elements, and highly non-local hydrogen bonds between the N- and C-terminal strands. CFr is known to form extremely stable homodimers, which retain their secondary structure till very high temperatures like 371 K and high concentrations of denaturants [59].

In [13,14], an earlier version of our model was used to study the folding of CFr. The simulations pointed to an unexpected folding mechanism. The N-terminal strand initially folds as a non-native continuation of the adjoining *α*-helix. After the other secondary structure elements form and diffuse to an approximately correct tertiary organization, the non-native extension of the helix unfolds and frees the N-terminal residues. These residues then attach to an existing *β*-hairpin to complete the three-stranded *β*-sheet of the native structure. Premature fastening of the chain ends in *β*-sheet contacts puts the molecule in a deep local energy minimum, in which the folding and proper arrangement of the other secondary structure elements is hampered by large steric barriers. The above "caching" mechanism, spontaneously emerging in the simulations, accelerates folding by helping the molecule avoid such local minima.

The folding properties of CFr, including the above mentioned caching mechanism, are preserved under the current modifications of the interaction potential. The centre of the native free-energy minimum shifts from bRMSD (all residues) of 1.7 Å as reported in [13] to about 2.2 Å. This state remains the minimum energy state, although the new energy function changes the energy ordering of the other low energy states. The runs made for this study (see Table 6) found 22 independent folding events. The free-energy landscape observed in the simulations is rather complex with a plethora of deep local minima sharing one or more secondary structure elements with the native structure. They differ in the registry and ordering of strands and the length of the helix. Longer runs are required for the MC simulations to correctly weight these different minima. Temperature dependence of the properties of CFr can therefore not be reliably obtained from these runs.

We note that the simulations ran on twice as many processors but were only about one sixth the length of those used for [13], in which 15 independent folding events were found. The improved efficiency is partly due to the changes in the energy function presented here, and partly due to the optimization of the parallel tempering described in [26].

### 3.9 GS-*α*_3 _W

GS-*α*_3 _W is a designed three-helix-bundle protein with 67 residues [60], whose structure was characterized by NMR [61]. The stability was estimated to be 4.6 kcal/mol in aqueous solution at 298 K, based on CD data [60].

**Figure 9:**
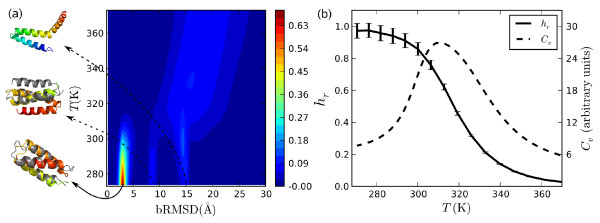
**The three-helix-bundle protein GS-*α*_3 _W**. (a) Variation of histogram of bRMSD with temperature. At high temperatures, there is a broad distribution of bRMSD with values > 10 Å. At lower temperatures there are three clearly separated clusters. Representative structures from these clusters are also shown (color) aligned with the native structure (gray). (b) Temperature dependence of specific heat, *C*_*v*_, and the ratio *h*_*r *_of the observed helix content and the helix content of the native structure.

It turns out that this protein is very easy to fold with our model. Our results are based on extensive sampling of the conformation space with 64 × 3.5 × 10^9 ^Monte Carlo updates, resulting in about 800 independent folding events to the native state. For this estimate, structures with bRMSD (all residues) under 5 Å were taken to be in the native minimum (see Fig. 9 for justification). Two visits to the native state were considered statistically independent (i) if they occurred in independent Markov chains, or (ii) if the two visits to the native state were separated by at least one visit to the highest temperature in the simulation. For the entire run, we spent about 10 days of computing time on 64 AMD Opteron processors running at 2.0 GHz.

In Fig. 9a, we show how the probabilities for structures with different bRMSD vary with temperature in the simulations. Clearly, the protein makes a transition from a rather continuous distribution of bRMSD at high temperatures to a distribution dominated by three well separated clusters. Analysis of the structures at the lower temperatures shows that all three free-energy minima consist almost exclusively of structures with all three helices of GS-*α*_3 _W formed. The plot of the ratio of the observed helix content and the helix content of the native state, shown in Fig. 9b, further supports this idea. The average value of this ratio approaches 1 as the temperature decreases below 300 K. The specific heat curve, also shown in Fig. 9b, indicates that the formation of these structures correlates with the steepest change in energy.

The cluster with a center at bRMSD ~3 Å dominates at the lowest temperatures. The structures contributing to the cluster with ~8–9 Å bRMSD superficially look like well folded three-helix bundles. But as illustrated in the figure, the arrangement of the helices is topologically distinct from the native arrangement. The cluster seen at larger bRMSD values is broader and consists of a host of structures in which two of the helices make a helical hairpin, but the third helix is not bound to it. The unbound helix could be at either side of the chain.

According to our model therefore, the population at the lowest temperatures consists of ~80% genuinely native structures, ~10% three-helix bundles with wrong topology, and ~10% other structures with as much helix content as the native state. In order to experimentally determine the true folded population of the protein, the experimental probe must be able to distinguish the native fold from the other helix rich structures described here.

## 4 Discussion

The model presented here is intrinsically fast compared to many other all-atom models, because all interactions are short range. By exploiting this property and using efficient MC techniques, it is possible to achieve a high sampling efficiency. We could, for example, generate more than 800 independent folding events for the 67-residue GS-*α*_3 _W. The speed of the simulations thus permits statistically accurate studies of the global free-energy landscape of peptides and small proteins.

In developing this potential, a set of 17 peptides with 10–37 residues was studied. The peptides were added to this set one at a time. To fold a new sequence sometimes required fine-tuning of the potential, sometimes not. A change was accepted only after testing the new potential on all previous sequences in the set. In its final form, the model folds all 17 sequences to structures similar to their experimental structures, for one and the same choice of potential parameters.

Also important is the stability of the peptides. A small polypeptide chain is unlikely to be a clear two-state folder, and therefore its apparent folded population will generally depend on the observable studied. For *β*-sheet peptides, we used the hydrophobicity energy *E*_hp _and the hydrogen bond-based nativeness measure *q*_hb _to monitor the melting behavior. The extracted *T*_m _values indeed showed a clear probe dependence; the *E*_hp_-based value was always larger than that based on *q*_hb_. For the *β*-hairpins studied, we found a good overall agreement between our *E*_hp_-based results and experimental data. For the three-stranded *β*-sheets, instead, the *q*_hb _results agreed best with experimental data. The reason for this difference is unclear. One contributing factor could be that interactions between aromatic residues play a more important role for the *β*-hairpins studied here than for the three-stranded *β*-sheets. These interactions may influence spectroscopic signals and are part of *E*_hp_. Probe-dependent *T*_m _values have also been obtained experimentally, for example, for trpzip2 [53].

The probe dependence makes the comparison with experimental data less straightforward. Nevertheless, the results presented clearly show that the model captures many experimentally observed stability differences. In particular, among related peptides, the calculated order of increasing thermal stability generally agrees with the experimental order, independent of which of our observables we use.

It is encouraging that the model is able to fold these 17 sequences. However, there is no existing model that will fold all peptides, and our model is no exception. Two sequences that we unsuccessfully tried to fold are the *β*-hairpins trpzip4 and U_16_, both with 16 residues. Trpzip4 is a triple mutant of GB1p with four tryptophans [52]. For trpzip4, our minimum energy state actually corresponded to the NMR-derived native state [52], but the population of this state remained low at the lowest temperature studied (~14% at 279 K, as opposed to an estimated *T*_m _of 343 K in experiments [52]). U_16 _is derived from the N-terminal *β*-hairpin of ubiquitin [62]. It has a shortened turn and has been found to form a *β*-hairpin with non-native registry [62]. In our simulations, this state was only weakly populated (~8% at 279 K, as opposed to an estimated ~80% at 288 K [62]). Instead, the main free-energy minima corresponded to the two *β*-hairpin states with the registry of native ubiquitin, one with native hydrogen bonds and the other with the complementary set of hydrogen bonds.

Our calibration of the potential relies on experimental data with non-negligible uncertainties, on a limited number of peptides. It is not evident that this potential will be useful for larger polypeptide chains. Therefore, as a proof-of-principle test, we also studied three larger systems, with very good results. Our simulations showed that, without having to adjust any parameter, the model folds these sequences to structures consistent with experimental data. Having verified this, it would be interesting to use the model to investigate the mechanisms by which these systems self-assemble, but such an analysis is beyond the scope of this article. The main purpose of our present study of these systems was to demonstrate the viability of our calibration approach.

The potential can be further constrained by confronting it with more accurate experimental data and data on new sequences. The challenge in this process is to ensure backward compatibility – new constraints should be met without sacrificing properties already achieved.

## 5 Conclusion

We have described and tested an implicit solvent all-atom model for protein simulations. The model is computationally fast and yet able to capture structural and thermodynamic properties of a diverse set of sequences. Its computational efficiency greatly facilitates the study of folding and aggregation problems that require exploration of the full free-energy landscape. A program package, called PROFASI [28], for single- and multi-chain simulations with this model is freely available to academic users.
